# The Relevance in the Neutrophil to Lymphocyte Ratio and the SARC-F Score in Gastrointestinal Diseases

**DOI:** 10.3390/jcm11072012

**Published:** 2022-04-03

**Authors:** Eiki Yamasaki, Hiroki Nishikawa, Masahiro Goto, Masahiro Matsui, Akira Asai, Kosuke Ushiro, Takeshi Ogura, Toshihisa Takeuchi, Shiro Nakamura, Kazuki Kakimoto, Takako Miyazaki, Shinya Fukunishi, Hideko Ohama, Keisuke Yokohama, Hidetaka Yasuoka, Kazuhide Higuchi

**Affiliations:** 1 The Second Department of Internal Medicine, Osaka Medical and Pharmaceutical University, Takatsuki 569-8686, Japan; eiki.yamasaki@ompu.ac.jp (E.Y.); masahiro.goto@ompu.ac.jp (M.G.); masa1987_11_18@yahoo.co.jp (M.M.); akira.asai@ompu.ac.jp (A.A.); ushiro.1989@icloud.com (K.U.); takeshi.ogura@ompu.ac.jp (T.O.); toshihisa.takeuchi@ompu.ac.jp (T.T.); shiro.nakamura@ompu.ac.jp (S.N.); kazuki.kakimoto@ompu.ac.jp (K.K.); takako.miyazaki@ompu.ac.jp (T.M.); shinya.fukunishi@ompu.ac.jp (S.F.); hideko.ohama@ompu.ac.jp (H.O.); hammer_0906@yahoo.co.jp (K.Y.); yh0403.4351@gmail.com (H.Y.); kazuhide.higuchi@ompu.ac.jp (K.H.); 2 The Premier Departmental Research of Medicine, Osaka Medical and Pharmaceutical University, Takatsuki 569-8686, Japan

**Keywords:** neutrophil to lymphocyte ratio, SARC-F, sarcopenia, gastrointestinal disease, correlation

## Abstract

We sought to clarify the relevance in the neutrophil to lymphocyte ratio (NLR) and the SARC-F score in patients with gastrointestinal diseases (G-Ds, *n* = 672, median age = 73 years). Univariate and multivariate analysis for the SARC-F score were performed. Advanced malignancy was identified in 162 patients (24.1%). The median of NLR for all cases was 2.65. The median of NLR in ECOG-PS 0 (*n* = 436), 1 (*n* = 128), 2 (*n* = 49) and 3 or 4 (*n* = 59) was 2.26, 2.97, 4.41 and 5.99 (overall *p* < 0.0001). NLR had a significant correlation with the SARC-F score (*r* = 0.54, *p* < 0.0001). The median of NLR in the SARC-F score ≥4 (recommended value for sarcopenia, *n* = 84) and <4 (*n* = 588) was 5.87 and 2.48 (*p* < 0.0001). In all subgroup analyses, similar trends were seen. In the multivariate analysis, ECOG-PS (*p* < 0.0001) and NLR (*p* < 0.0001) were independent factors, while age had a trend for significance (*p* = 0.0686). In conclusion, we would like to emphasize the usefulness of NLR, a simple marker assessed only by blood tests, in predicting the possibility for sarcopenia by the SARC-F in G-Ds.

## 1. Introduction

Sarcopenia, defined by a decrease in the quantity and quality of skeletal muscle, can be broadly divided into primary sarcopenia, which is associated with aging, and secondary sarcopenia, which is due to malnutrition, physical inactivity, and disease burden itself [1,2,3,4,5,6,7,8,9]. Patients with gastrointestinal diseases (G-Ds) are prone to secondary sarcopenia due to a combination of increased protein catabolism caused by an increased inflammatory response, metabolic abnormalities, and malnutrition caused by poor dietary intake [10,11,12]. On the other hand, SARC-F, which is a screening method on sarcopenia, is a questionnaire with five questions [13,14,15]. Subjects reply to these questions on a scale of 0 to 2, and the total points are assessed (possible range: 0–10 points) [14]. Subjects with a SARC-F score of four points or more are regarded as quite likely being cases of sarcopenia [14]. The higher SARC-F score can be associated with reduced physical function [16,17]. The latest international judging standards on sarcopenia recommend the use of SARC-F as a first screening method on sarcopenia [18,19]. We have reported that elevated SARC-F score can be an adverse predictor in patients with advanced gastrointestinal malignancy [11].

White blood cells are classified into neutrophils, lymphocytes, basophils, eosinophils, and monocytes. Neutrophils and lymphocytes are used not only as indicators of immune function, but also as indicators of inflammation. Neutrophils are mainly involved in acute phase inflammation, while lymphocytes are mainly involved in chronic phase inflammation [20]. The neutrophil to lymphocyte ratio (NLR) has been shown to be a helpful prognostic marker in advanced malignancies, and a recent meta-analysis has reported that elevated NLR is linked to an adverse outcome in numerous solid malignancies [21]. Neutrophils play a significant role in the production of (1) ligands that promote malignant cell proliferation and invasion and (2) cytokines that promote angiogenesis. An increase in neutrophils is therefore closely related to tumor growth and metastasis [22]. On the other hand, lymphocytes are responsible for the immune function of the host, and a decrease in lymphocytes can damage the host’s anti-tumor immunity and worsen the prognosis [23]. Thus, in advanced cancer, NLR reflects the balance between tumor promotion and anti-tumor immune status [24,25].

However, as far as we are aware, there have been no reports regarding the relevance in the NLR and the SARC-F score in patients with G-Ds. We have decided to conduct the current research because we believe that these problems need to be solved.

## 2. Patients and Methods

### 2.1. Patients and Our Study

In our department, we have asked each hospitalized patient to respond to the SARC-F questionnaire and to test grip strength (GS) on admission. From October 2020 until November 2021, there were 672 Japanese G-D individuals having both SARC-F score and data for GS in our medical record. Blood test results were the results on admission. First, the relevance in the NLR and baseline features such as ECOG-PS and the SARC-F score was explored. Univariate and multivariate analysis for the SARC-F score were subsequently performed. The analyzed factors in the univariate and multivariate analysis were continuous parameters. We defined advanced cancer as stage III or severer cancer. Decrease in GS was defined as <28 kg (male) and <18 kg (female) as recommended elsewhere [18]. The SARC-F score ≥4 was decided to involve highly possibility for sarcopenia [18]. The relevant ethics committee of our hospital gave an approval of ethics (approval number, 2021-165). 

### 2.2. Statistical Procedure

In analyzing continuous variables, we selected the appropriate method among Student’s *t* test, Mann–Whitney *U* test or Pearson correlation coefficient *r* for comparing 2 groups, and also selected the appropriate method between ANOVA and Kruskal–Wallis test for comparing multiple groups. Variables with statistically significant correlation with the SARC-F score were entered into a multivariate regression analysis by the least square method, and candidate parameters were finally selected. A continuous variable was shown as a median with interquartile range (IQR). A *p* value of 0.05 was a threshold for significance by the JMP ver. 15 (SAS Institute Inc., Cary, NC, USA).

## 3. Results

### 3.1. Patient Baseline Features

Baseline data (at the time of admission) for all analyzed subjects (*n* = 672, 415 males and 257 females, median (IQR) age = 73 (63–79) years) are summarized in Table 1. The median (IQR) of body mass index (BMI) was 22.0 (19.6–24.4) kg/m^2^ (missing data, *n* = 2). ECOG-PS 0 was seen in 436 patients (64.9%), 1 in 128 (19.0%), 2 in 49 (7.3%), 3 in 41 (6.1%) and 4 in 18 (2.7%). Upper gastrointestinal disease (U-G-D) was seen in 161 patients (advanced malignancy, 39 cases (24.2%)), lower gastrointestinal disease (L-G-D) in 178 (advanced malignancy, 30 cases (16.9%)), biliary and pancreatic disease (BP-D) in 236 (advanced malignancy, 65 cases (27.5%)) and liver disease (L-D) in 97 (advanced malignancy, 28 cases (28.9%)). Overall, advanced malignancy was identified in 162 patients (24.1%). Patients with conditions other than advanced cancer included early stage cancer and benign diseases such as benign polyp, biliary tract benign diseases, pancreatitis, inflammatory bowel diseases such as ulcerative colitis, gastrointestinal bleeding lesion, infectious diseases, etc. The median (IQR) of the SARC-F score was 0 (0–2). SARC-F score 0 was seen in 388 cases (57.7%), 1 in 105 (15.6%), 2 in 57 (8.5%), 3 in 38 (5.7%) and ≥4 in 84 (12.5%). The median (IQR) of GS in male and female was 28.9 (23.6–34.2) kg and 17.0 (13.2–20.4) kg. The proportion of decrease in GS in male (<28 kg) and female (<18 kg) was 44.6% (185/415) in male and 56.4% (145/257) in female.

### 3.2. The NLR according to ECOG-PS, Anatomical Category of Disease and BMI

**Figure 1:**
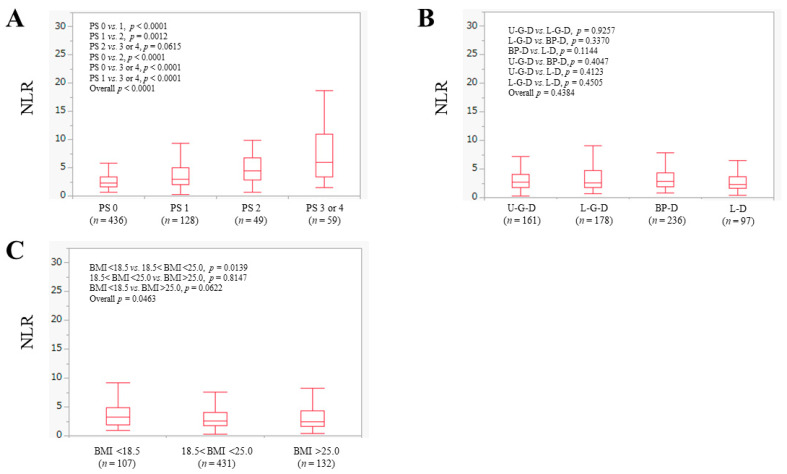
The neutrophil to lymphocyte ratio (NLR) according to (**A**) ECOG-PS, (**B**) anatomical category of disease, and (**C**) body mass index (BMI, kg/m^2^) in all cases (*n* = 672). U-G-D, upper gastrointestinal disease; L-G-D, lower gastrointestinal disease; BP-D, biliary and pancreatic disease; and L-D, liver disease.

The median (IQR) of NLR for all cases was 2.65 (1.79–4.31). The median (IQR) of NLR in ECOG-PS 0 (*n* = 436), 1 (*n* = 128), 2 (*n* = 49) and 3 or 4 (*n* = 59) was 2.26 (1.66–3.36), 2.97 (2.0–4.98), 4.41 (2.85–6.78) and 5.99 (3.33–10.92) (ECOG-PS 0 vs. 1, *p* < 0.0001; ECOG-PS 1 vs. 2, *p* = 0.0012; ECOG-PS 2 vs. 3 or 4, *p* = 0.0615; ECOG-PS 0 vs. 2, *p* < 0.0001; ECOG-PS 0 vs. 3 or 4, *p* < 0.0001; ECOG-PS 1 vs. 3 or 4, *p* < 0.0001; overall *p* < 0.0001) (Figure 1A).

The median (IQR) of NLR in U-G-D (*n* = 161), L-G-D (*n* = 178), BP-D (*n* = 236) and L-D (*n* = 97) was 2.65 (1.75–4.0), 2.54 (1.76–4.77), 2.82 (1.93–4.37) and 2.32 (1.66–3.63) (U-G-D vs. L-G-D, *p* = 0.9257; L-G-D vs. BP-D, *p* = 0.3370; BP-D vs. L-D, *p* = 0.1144; U-G-D vs. BP-D, *p* = 0.4047; U-G-D vs. L-D, *p* = 0.4123; L-G-D vs. L-D, *p* = 0.4505; overall *p* = 0.4384) (Figure 1B).

Our cohort was divided into three categories according to the baseline BMI. The median (IQR) of NLR in patients with BMI < 18.5 kg/m^2^ (*n* = 107), 18.5 kg/m^2^ < BMI < 25 kg/m^2^ (*n* = 431) and BMI > 25 kg/m^2^ (*n* = 132) was 3.21 (1.96–4.90), 2.59 (1.78–4.09) and 2.48 (1.66–4.38) (BMI < 18.5 kg/m^2^ vs. 18.5 kg/m^2^ < BMI < 25 kg/m^2^, *p* = 0.0139; 18.5 kg/m^2^ < BMI < 25 kg/m^2^ vs. BMI > 25 kg/m^2^, *p* = 0.8147; BMI < 18.5 kg/m^2^ vs. BMI > 25 kg/m^2^, *p* = 0.0622; overall *p* = 0.0463) (Figure 1C).

### 3.3. The Relevance in the NLR and the SARC-F Score

**Figure 2:**
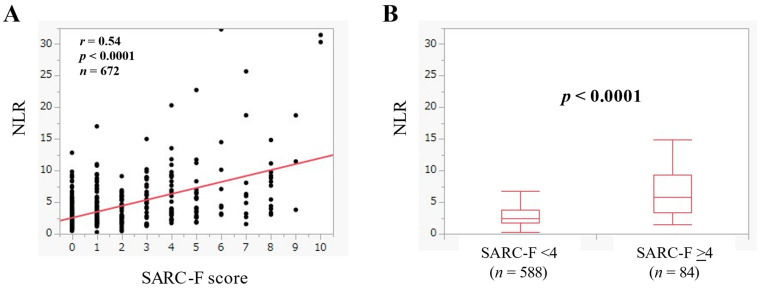
(**A**) The relevance in the NLR and the SARC-F score in all cases. (**B**) Comparison of NLR between SARC-F <4 (*n* = 588) and ≥4 (*n* = 84) in all cases.

NLR had a significant correlation with the SARC-F score (*r* = 0.54, *p* < 0.0001) (Figure 2A). The median (IQR) of NLR in the SARC-F score ≥4 (*n* = 84) and <4 (*n* = 588) was 5.87 (3.35–9.41) and 2.48 (1.69–3.73) (*p* < 0.0001) (Figure 2B).

### 3.4. The Relevance in the NLR and the SARC-F Score according to the Anatomical Category

**Figure 3:**
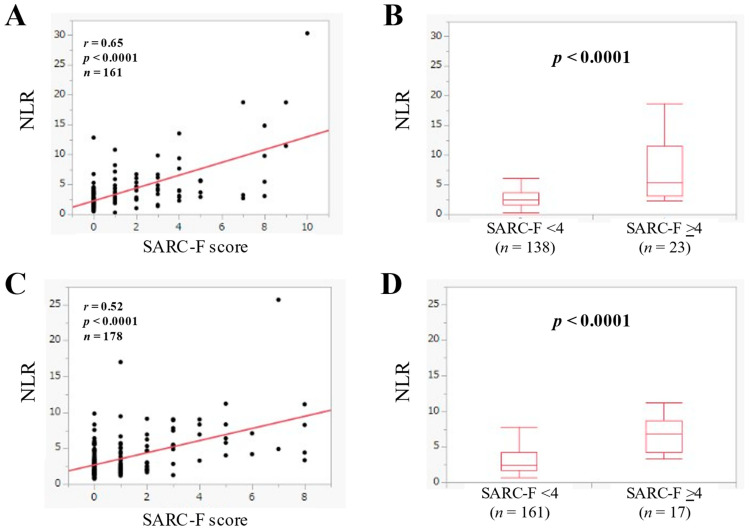
(**A**) The relevance in the NLR and the SARC-F score in U-G-D cases (*n* = 161). (**B**) Comparison of NLR between SARC-F <4 (*n* = 138) and ≥4 (*n* = 23) in U-G-D cases. (**C**) The relevance in the NLR and the SARC-F score in L-G-D cases (*n* = 178). (**D**) Comparison of NLR between SARC-F <4 (*n* = 161) and ≥4 (*n* = 17) in L-G-D cases.

In U-G-D (*n* = 161), NLR had a significant correlation with the SARC-F score (*r* = 0.65, *p* < 0.0001). (Figure 3A) In U-G-D, the median (IQR) of NLR in the SARC-F score ≥4 (*n* = 23) and <4 (*n* = 138) was 5.43 (3.07–11.44) and 2.42 (1.63–3.64) (*p* < 0.0001) (Figure 3B).

In L-G-D (*n* = 178), NLR had a significant correlation with the SARC-F score (*r* = 0.52, *p* < 0.0001) (Figure 3C). In L-G-D, the median (IQR) of NLR in the SARC-F score ≥4 (*n* = 17) and <4 (*n* = 161) was 6.91 (4.29–8.67) and 2.40 (1.69–4.26) (*p* < 0.0001) (Figure 3D).

**Figure 4:**
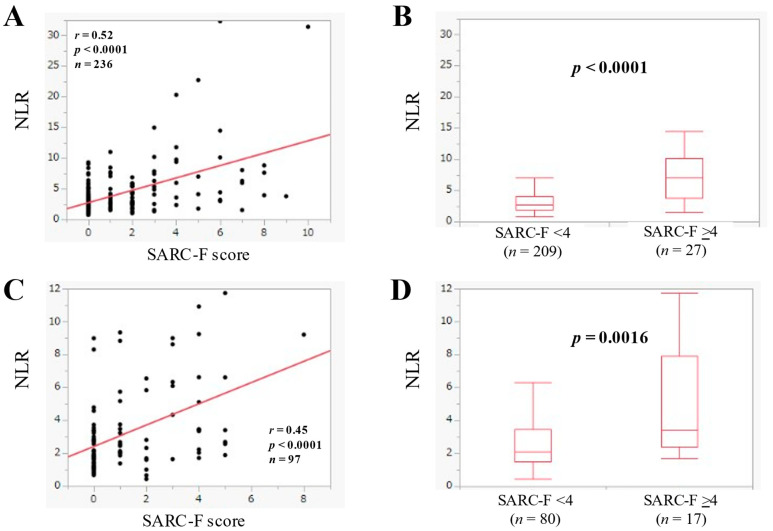
(**A**) The relevance in the NLR and the SARC-F score in BP-D cases (*n* = 236). (**B**) Comparison of NLR between SARC-F <4 (*n* = 209) and ≥4 (*n* = 27) in BP-D cases. (**C**) The relevance in the NLR and the SARC-F score in L-D cases (*n* = 97). (**D**) Comparison of NLR between SARC-F <4 (*n* = 80) and ≥4 (*n* = 17) in L-D cases.

In BP-D (*n* = 236), NLR had a significant correlation with the SARC-F score (*r* = 0.52, *p* < 0.0001) (Figure 4A). In BP-D, the median (IQR) of NLR in the SARC-F score ≥4 (*n* = 27) and <4 (*n* = 209) was 7.0 (3.81–10.13) and 2.65 (1.84–4.01) (*p* < 0.0001) (Figure 4B).

In L-D (*n* = 97), NLR had a significant correlation with the SARC-F score (*r* = 0.45, *p* < 0.0001) (Figure 4C). In L-D, the median (IQR) of NLR in the SARC-F score ≥4 (*n* = 17) and <4 (*n* = 80) was 3.41 (2.40–7.91) and 2.09 (1.51–3.45) (*p* = 0.0016) (Figure 4D).

### 3.5. The Relevance in the NLR and the SARC-F Score in Patients with Advanced Cancer

**Figure 5:**
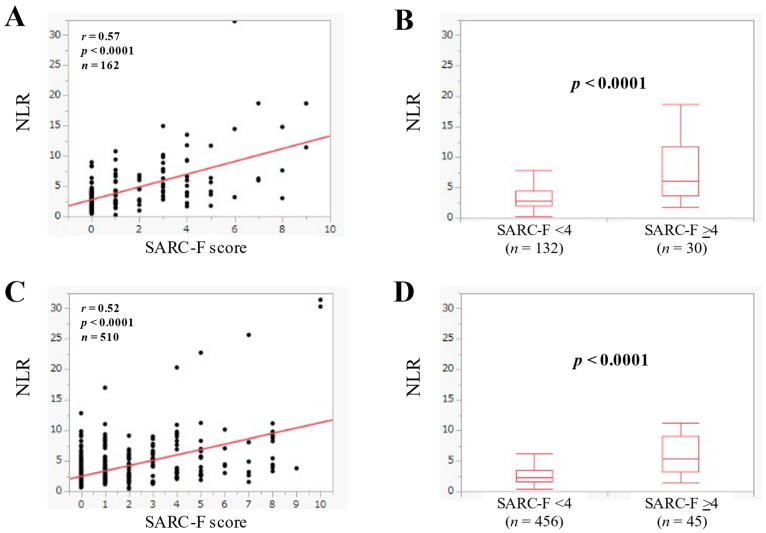
(**A**) The relevance in the NLR and the SARC-F score in advanced cancer cases (*n* = 162). (**B**) Comparison of NLR between SARC-F <4 (*n* = 132) and ≥4 (*n* = 30) in advanced cancer cases. (**C**) The relevance in the NLR and the SARC-F score in non-advanced cancer cases (*n* = 510). (**D**) Comparison of NLR between SARC-F <4 (*n* = 456) and ≥4 (*n* = 45) in non-advanced cancer cases.

The median (IQR) of NLR in advanced cancer (*n* = 162) was 3.33 (2.25–5.69), and NLR was significantly correlated with the SARC-F score (*r* = 0.57, *p* < 0.0001) (Figure 5A). In advanced cancer, the median (IQR) of NLR in the SARC-F score ≥4 (*n* = 30) and <4 (*n* = 132) was 6.14 (3.60–11.75) and 2.91 (2.04–4.43) (*p* < 0.0001) (Figure 5B).

### 3.6. The Relevance in the NLR and the SARC-F Score in Patients without Advanced Cancer

The median (IQR) of NLR in patients without advanced cancer (*n* = 510) was 2.49 (1.71–4.0), and NLR significantly had a significant correlation the SARC-F score (*r* = 0.52, *p* < 0.0001) (Figure 5C). In patients without advanced cancer, the median (IQR) of NLR in the SARC-F score ≥4 (*n* = 54) and <4 (*n* = 456) was 5.48 (3.26–9.07) and 2.32 (1.66–3.51) (*p* < 0.0001) (Figure 5D).

### 3.7. The Relevance in the NLR and GS

**Figure 6:**
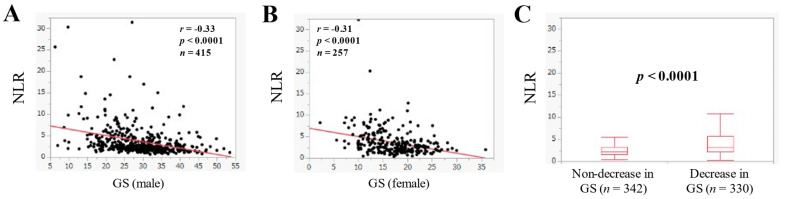
The correlation in the NLR and grip strength (GS) in male ((**A**), *n* = 415) and female ((**B**), *n* = 257). (**C**) Comparison of NLR in patients with non-decrease in GS (*n* = 342) and decrease in GS (*n* = 330).

In males, NLR was significantly correlated with GS (*r* = −0.33, *p* < 0.0001) (Figure 6A). Likewise, in females, NLR was significantly correlated with GS (*r* = −0.31, *p* < 0.0001) (Figure 6B). NLR in patients with decrease in GS (*n* = 330, median (IQR) = 3.31 (2.23–5.69)) was significantly higher than that without decrease in GS (*n* = 342, median (IQR) = 2.14 (1.58–3.21) (*p* < 0.0001) (Figure 6C).

### 3.8. Univariate and Multivariate Analysis of Factors Linking to the SARC-F Score

In the univariate analysis of factors linking to the SARC-F score, age, ECOG-PS, hemoglobin, serum albumin, NLR, C reactive protein (CRP) and estimated glomerular filtration rate were significant factors (Table 2). In the multiple regression analysis (multivariate analysis), ECOG-PS and NLR were independent factors linking to the SARC-F score, while age had a trend for significance (*p* = 0.0686) (Table 3).

## 4. Discussion

Sarcopenia is at the central part of physical frailty, and frailty control is one of the health measure projects promoted by the Ministry of Health, Labor and Welfare in Japan [5]. The number of published papers on sarcopenia research has been also rapidly increasing worldwide [5]. In recent years, the Asian and European sarcopenia assessment criteria have been revised one after another, and the use of the SARC-F (i.e., screening method) has been recommended in the revised guidelines [18,19]. On the other hand, the close relevance in the NLR and prognosis has been reported in various malignancies in recent years. Although the mechanism is still unclear, it has been implied that the cancer-associated inflammatory microenvironment is involved in the process of cancer progression. Neutrophils are involved in tumor promotion, while lymphocytes play a role in anti-tumor immunity. Therefore, elevated NLR may reflect tumor growth and progression, and may be a predictive marker of poor prognosis [26]. NLR is attracting attention as one of the most sensitive indicators of inflammatory status not only in the field of oncology, but also in the fields of cardiovascular disease, diabetes, and infectious diseases [27,28,29]. Recently, the usefulness of NLR in assessing the severity of COVID-19 infection and determining treatment response has also been reported [30,31,32,33]. NLR is inexpensive to measure, can be measured at any facility, and can be measured frequently in daily practice, making it more valuable and clinically applicable than existing markers that are complicated to measure. However, reports on the relevance in the NLR and SARC-F in patients with G-Ds are scarce. Therefore, the results of this study, which analyzed a large number of cases (*n* = 672), are significant and worth reporting.

The mean value of NLR in healthy subjects has been reported to be 1.65 [34], and our median NLR in the present study was 2.65. In patients with G-Ds, NLR may be elevated due to increased inflammation and decreased immune status. The present study showed a good correlation between the NLR and the SARC-F score in the overall cases and all subgroups, and the relationship between GS and the NLR was similar. These results suggest that NLR can be a marker that well reflects the severity of sarcopenia. Although CRP is a marker of acute inflammation, it was not a significant factor in the multivariate analysis. While NLR is a complex marker of inflammatory and immune status, and the difference in the nature of CRP and NLR as inflammatory markers may have affected the present results. NLR has been reported to be unaffected by cytokines that affect CRP [35]. ECOG-PS was extracted as an independent factor, and a significant positive correlation was seen in ECOG-PS and the NLR (*r* = 0.48, *p* < 0.0001). As mentioned earlier, the NLR is easy to measure and correlates well with the SARC-F score and ECOG-PS, so this marker appears to be useful in the daily clinical practice. It is worth mentioning that the strong correlation between ECOG-PS and the SARC-F score (*r* = 0.79) was found, although it is a little beyond the purpose of this study. The NLR has been reported to be elevated mainly in advanced cancers, and in this study, the median of NLR in advanced cancer cases and non-advanced cancer cases was 3.33 and 2.49 (*p* < 0.0001), which is consistent with previous reports [21]. The group with BMI < 18.5 kg/m^2^ showed a tendency to have higher NLR. The median BMI of patients with advanced and non-advanced cancer (21.3 kg/m^2^ vs. 22.2 kg/m^2^, *p* = 0.0160) may also have influenced the current results. Age showed a significant trend in the multivariate analysis. It can be inferred that the development of sarcopenia in G-Ds is a complex interplay of aging and disease pathogenesis. It may also be necessary to evaluate whether primary or secondary factors are more strongly involved in sarcopenia in G-Ds [6].

It is necessary to point out several limitations of this study besides the fact that this study was a single-center and retrospective observational study. Firstly, as data for skeletal muscle mass in our cohort were missing, it is possible that we were missing cases of sarcopenia that are included among cases with a SARC-F score of less than 4. Secondly, the present cohort included a lot of types of G-Ds. Thirdly, in this study, data at the time of admissions were used, which may lead to bias, especially in patients with infectious diseases where data fluctuate widely. Therefore, meticulous care should be paid on interpreting the study results. Nevertheless, our study results implied that NLR in patients with G-Ds well correlates with the SARC-F score. Finally, we would like to emphasize the usefulness of NLR, a simple marker that can be assessed only by blood tests, in predicting the possibility for sarcopenia by the SARC-F in patients with G-Ds.

## Figures and Tables

**Table 1:** Baseline characteristics (*n* = 672).

	*n* or Median (IQR)
Age (years)	73 (63–79)
Gender, male/female	415/257
ECOG-PS, 0/1/2/3/4	436/128/49/41/18
Anatomical disease type	
Upper gastrointestinal disease	161
Lower gastrointestinal disease	178
Biliary and pancreatic disease	236
Liver disease	97
Advanced cancer, yes/no	162/510
Body mass index (kg/m^2^)	22.0 (19.6–24.4)
Alanine aminotransferase (IU/L)	19 (12–32)
C reactive protein (mg/dL)	0.18 (0.06–0.94)
eGFR (ml/min/1.73 m^2^)	67 (55–81)
Serum albumin (g/dL)	3.8 (3.4–4.2)
Hemoglobin (g/dL)	12.5 (11.1–13.8)
Platelet count (×10^4^/μL)	22.0 (16.7–27.9)
White blood cell (/μL)	5910 (4768–7693)
Neutrophil count (/μL)	3706 (2809–5408)
Total lymphocyte count (/μL)	1397 (1069–1842)
Neutrophil to lymphocyte ratio	2.65 (1.79–4.31)
SARC-F score	0 (0–2)
Grip strength (male, kg)	28.9 (23.6–34.2)
Grip strength (female, kg)	17.0 (13.2–20.4)

IQR, interquartile range; eGFR, estimated glomerular filtration rate.

**Table 2:** Correlation in the SARC-F score and baseline characteristics.

	*r*	*p* Value
Age	0.25	<0.0001
BMI	−0.03	0.3967
ECOG-PS	0.79	<0.0001
ALT	−0.009	0.8207
Hemoglobin	−0.27	<0.0001
Platelet count	0.03	0.4707
Serum albumin	−0.30	<0.0001
NLR	0.54	<0.0001
CRP	0.21	<0.0001
eGFR	−0.10	0.0074

BMI, body mass index; ALT, alanine aminotransferase; NLR, neutrophil to lymphocyte ratio; CRP, C reactive protein; eGFR, estimated glomerular filtration rate.

**Table 3:** Multivariate analysis (multiple regression analysis) of factors linking to the SARC-F score.

	Estimates	Standard Error	*p* Value
Age	0.0066012	0.003619	0.0686
ECOG-PS	1.2992242	0.050599	<0.0001
Hemoglobin	−0.017435	0.025668	0.4972
Serum albumin	0.042363	0.095874	0.6587
NLR	0.1257596	0.016222	<0.0001
CRP	−0.009757	0.012562	0.4376
eGFR	−0.001313	0.002261	0.5615

NLR, neutrophil to lymphocyte ratio; CRP, C reactive protein; eGFR, estimated glomerular filtration rate.

## Data Availability

The data are not publicly available due to personal information of our study cohort.

## Abbreviation

G-D, gastrointestinal disease; NLR, neutrophil to lymphocyte ratio; GS, grip strength; IQR, interquartile range; BMI, body mass index; U-G-D, upper gastrointestinal disease; L-G-D, lower gastrointestinal disease; BP-D, biliary and pancreatic disease; L-D, liver disease; CRP, C reactive protein.
